# Effects of Cadmium on ZO-1 Tight Junction Integrity of the Blood Brain Barrier

**DOI:** 10.3390/ijms20236010

**Published:** 2019-11-29

**Authors:** Jacopo Junio Valerio Branca, Mario Maresca, Gabriele Morucci, Tommaso Mello, Matteo Becatti, Luigia Pazzagli, Ilaria Colzi, Cristina Gonnelli, Donatello Carrino, Ferdinando Paternostro, Claudio Nicoletti, Carla Ghelardini, Massimo Gulisano, Lorenzo Di Cesare Mannelli, Alessandra Pacini

**Affiliations:** 1 Department of Experimental and Clinical Medicine, Anatomy and Histology Section, University of Florence, 50134 Florence, Italy; gabriele.morucci@unifi.it (G.M.); donatello.carrino@unifi.it (D.C.); ferdinando.paternostro@unifi.it (F.P.); claudio.nicoletti@unifi.it (C.N.); massimo.gulisano@unifi.it (M.G.); 2 Department of Neuroscience, Psychology, Drug Research and Child Health (NEUROFARBA), Pharmacology and Toxicology Section, University of Florence, 50139 Florence, Italy; mario.maresca@unifi.it (M.M.); carla.ghelardini@unifi.it (C.G.); lorenzo.mannelli@unifi.it (L.D.C.M.); 3 Department of Experimental and Clinical Biomedical Sciences “Mario Serio”, University of Florence, 50134 Florence, Italy; tommaso.mello@unifi.it (T.M.); matteo.becatti@unifi.it (M.B.); luigia.pazzagli@unifi.it (L.P.); 4 Department of Biology, Plant Ecology and Physiology Laboratory, University of Florence, 50121 Florence, Italy; ilaria.colzi@unifi.it (I.C.); cristina.gonnelli@unifi.it (C.G.)

**Keywords:** cadmium, blood brain barrier, RBE4 cell line, ZO-1, ROS production, ER stress, caspase-3 activation, ATP extrusion

## Abstract

Cadmium (Cd) is a highly toxic environmental pollutant released from the smelting and refining of metals and cigarette smoking. Oral exposure to cadmium may result in adverse effects on a number of tissues, including the central nervous system (CNS). In fact, its toxicity has been related to neurological disorders, as well as neurodegenerative diseases such as Alzheimer’s and Parkinson’s diseases. Under normal conditions, Cd barely reaches the brain in adults because of the presence of the blood–brain barrier (BBB); however, it has been demonstrated that Cd-dependent BBB alteration contributes to pathogenesis of neurodegeneration. However, the mechanism underlying Cd-dependent BBB alteration remain obscure. Here, we investigated the signaling pathway of Cd-induced tight junction (TJ), F-actin, and vimentin protein disassembly in a rat brain endothelial cell line (RBE4). RBE4 cells treated with 10 μM cadmium chloride (CdCl_2_) showed a dose- and time-dependent significant increase in reactive oxygen species (ROS) production. This phenomenon was coincident with the alteration of the TJ zonula occludens-1 (ZO-1), F-actin, and vimentin proteins. The Cd-dependent ROS increase elicited the upregulation of GRP78 expression levels, a chaperone involved in endoplasmic reticulum (ER) stress that induces caspase-3 activation. Further signal profiling by the pannexin-1 (PANX1) specific inhibitor ^10^Panx revealed a PANX1-independent increase in ATP spillage in Cd-treated endothelial cells. Our results point out that a ROS-dependent ER stress-mediated signaling pathway involving caspase-3 activation and ATP release is behind the BBB morphological alterations induced by Cd.

## 1. Introduction

Cadmium (Cd) is one of the most harmful heavy metals and environmental pollutants widely spread in nature [1]. The main sources of human exposure to Cd range from occupational [2], to tobacco smoke, industrial emission, and contaminated food in the general population [3,4,5,6,7].

Cd toxicity is well known to affect a number of organ and tissues both at acute and chronic doses. The effect of Cd exposure is strictly dose-dependent: at high doses, Cd can progressively elicit cell injury, cell death, and organ failure, and at low doses it may modulate specific mechanisms without marked cellular toxicity [8].

Many in vivo and in vitro studies provide evidence that Cd induces neurotoxicity and damage to the peripheral nervous system (PNS) and central nervous system (CNS) [8,9,10,11,12,13] with a wide spectrum of clinical symptoms. They include neurological disturbances [14], neurodegenerative disorders such as Alzheimer’s disease (AD) and Parkinson’s disease (PD) [15], amyotrophic lateral sclerosis, multiple sclerosis [16,17], myalgic encephalomyelitis [18], olfactory dysfunction [19], peripheral neuropathy [20,21], mental retardation, and learning disabilities.

The CNS is protected from the entry of a number of potential toxicants by a continuous and highly selective cellular barrier, the blood–brain barrier (BBB), anatomically separating the sensitive parenchyma of the nervous system from the circulating blood.

Cadmium can enter the CNS either by bypassing the BBB through the olfactory pathway, especially in cigarette smokers and air pollution exposure [1,22], or by directly crossing the BBB via contaminated food and bloodstream [5,23].

Normal blood plasma cadmium concentrations in adults without excessive exposure are generally 8–30 nM [24,25], other authors consider 1.8–50 nM as the normal range [26]. Recently, the WHO [27] reported that the acceptable blood level of cadmium is 2–10 nM. Concentrations above 0.045 µM warrant careful investigation [28]. There are some discrepancies in the definition of toxic blood levels. In 1980, Kaye reported that the approximate lethal blood level is 4.5 µM [25]. A recent paper regarding the blood Cd levels of Nigerian subjects exposed to this metal showed that the mean blood value was 4 µM, without declared toxic effects, and it only had an “increased health risk” [29]. Acute poisoning with marked lethargy and fever was reported with a blood cadmium concentration of 0.25 µM [26].

In addition, it is worth underlining that Cd hardly reaches the nervous tissue under normal conditions because of the presence of the BBB; however, Cd can easily cross the immature BBB and accumulate in developing brains in young animals [30,31].

Moreover, the adult brain can also be affected by cadmium toxicity depending on the integrity of the BBB. Cd can increase BBB permeability in rats, penetrate and accumulate in the CNS, and lead to intracellular accumulation and cerebral damage [32,33,34]. In adult rats, high concentrations of Cd can accumulate in nervous tissue if co-exposed with a vehicle such as ethanol, as this vehicle can easily diffuse across biological membranes and allow Cd to also penetrate the BBB [35].

It is not clear if all the components constituting the BBB (i.e., neurons, astrocytes, and capillary endothelium) are equally sensitive to cadmium toxicity, but endothelial cells lining the luminal surface of brain blood vessels represent the first line of defense facing cadmium ions circulating in the bloodstream. An increased permeability or damage to the BBB endothelial layer can lead to uncontrolled passage of ions and proteins, allowing more Cd to enter the brain and, in turn, provoking severe neurotoxicity and impairment in neural tissue functioning [30].

Furthermore, in vitro data are quite different, and 0.5 µM is considered an ultra-low Cd concentration able to attenuate angiogenesis in both wound healing and chick chorioallantoic membrane (CAM) assays. In addition, the same concentration of Cd reduces bradykinin (BK), a powerful angiogenic agent, and mediates both tube formation in 3D matrigel matrix and ex vivo angiogenesis in CAM models, suggesting a protective role of Cd against tumor angiogenesis [36].

Based on this evidence, concentrations ranging from 1 to 100 µM (as those used in the present work) can be considered relevant to mimic cadmium-mediated damage of tissues or body compartments [37]. Moreover, based on previously reported data [38,39,40], we adopted the best experimental conditions that could better reflect an in vivo situation, mimicking chronic exposure conditions. For this reason, we chose the concentration of 10 μM that was lowly cytolytic but, at the same time, enabled us to study the Cd effects at the cellular level.

Nevertheless, the underlying mechanisms of Cd neurotoxicity are complex [41], and the detailed signal transduction pathways leading to BBB endothelial alterations are not completely elucidated.

In view of the above, in the present study, we hypothesized that the neurotoxic effects of Cd could be induced by a Cd-dependent alteration of the cell-to-cell junctional apparatus of the endothelial layer. Therefore, in the present study we investigated the effects of Cd on the ZO-1 tight junction (TJ) protein that anchors to the cytoskeletal apparatus [42].

Indeed, a characteristic feature of brain microvascular endothelial cells is the formation of tight junctions (TJs) sealing endothelial cells [43,44,45]. ZO-1 peripherally associated membrane proteins interact together in order to anchor other membrane proteins like claudins and occludins to the actin cytoskeleton [46,47,48]. Filamentous actin microfilament (F-actin) and the intermediate filament vimentin are the major constituents of the cytoskeleton that establishes inter-endothelial junctional integrity, defining the peripheral morphological belt of endothelial cells [49,50], and connecting the nuclear periphery to the inner surface of the plasma membrane [51]. For their characteristics, all these proteins are commonly used as markers of tight junction formation in monolayers of brain microvascular endothelial cells [44].

Furthermore, it is worth noting that a large volume of data points out that the neurotoxic effects of Cd ions are mainly mediated by the indirect production of high levels of reactive oxygen species (ROS) and induction of apoptosis [52,53]. On the other hand, the contribution of the endoplasmic reticulum (ER) stress response to oxidative stress in maintaining cell homeostasis and eventually triggering apoptosis signaling, as well as in playing a key role in Cd-induced mitochondrial-mediated apoptosis, has been recently demonstrated in different in vitro and in vivo studies [54,55,56].

Thus, given the notion mentioned above, we tested the expression of GRP78, a known unfolded protein response (UPR)-related chaperone [57,58,59,60,61]. Moreover, assuming that Cd-dependent BBB dysfunction is related to ATP extrusion as previously reported [62,63,64], we would also evaluate the role of PANX1, one of the putative channels mediating ATP spillage [65] and caspase-3 as a PANX1-openining channel mediator as previously reported [66].

Here, we identify that Cd induces a ROS-dependent ER stress-mediated signaling pathway involving caspase-3 activation that triggers ATP release by a PANX1 channel-independent mechanism, leading to structural morphologic alterations of the ZO-1 TJ component in a rat brain endothelial cell line (RBE4), a well-characterized and widely used BBB model [67,68].

## 2. Results

### 2.1. Accumulation of Cd and Its Effect on RBE4 Cell Viability

In this study, we utilized the immortalized RBE4 cells that express all the enzymes and transporters considered specific for the blood–brain barrier endothelium with similar characteristics to those expected from in vivo analyses [69,70,71,72,73]. Also, RBE4 cells are responsive to astroglial factors (i.e., astrocyte-derived angiopoietin-1, sonic hedgehog, and apolipoprotein E) [74].

First of all, by atomic absorption spectrophotometry, we analyzed total Cd accumulation in RBE4 cells incubated for 24 h in serum-free medium with 10 µM CdCl_2_, and Cd content was normalized to total dry weight (*n* = 3). Total Cd accumulation in RBE4 cells incubated with the metal was 224.3 ± 8.88 μg/g dry weight; background levels of Cd in untreated controls was 3.9 ± 0.37 μg/g dry weight (*n* = 3; *p* < 0.01).

**Figure 1:**
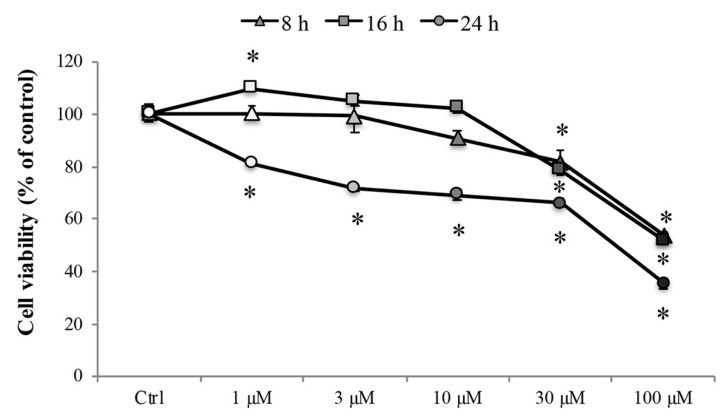
RBE4 cell viability. RBE4 cells (2.5 × 10^4^ cells/well) were incubated with CdCl_2_ (1–100 μM) for 8, 16, or 24 h. Viability was quantified by the 3-(4,5-di-methylthiazol-2-yl)-2,5-diphenyltetrazolium bromide (MTT) assay; absorbance was measured at 570 nm. Values are expressed in percentage of control absorbance as the mean ± S.E.M. of five independent experiments, *n* = 25. Control condition absorbance was fixed at 100%; * *p* < 0.05 vs. control (untreated cells).

Later, to investigate the effect of Cd on the cell viability, the 3-(4,5-di-methylthiazol-2-yl)-2,5-diphenyltetrazolium bromide (MTT) assay was performed after treatment with various concentrations of CdCl_2_ (1 to 100 µM) for 8, 16, and 24 h in RBE4 cells, considered relevant for mimicking Cd-mediated damage of tissues or body compartments [37]. As shown in Figure 1, treatment with CdCl_2_ decreased cell viability significantly (* *p* < 0.05 vs. control) in a concentration-dependent manner. Treatment with 30 and 100 μM CdCl_2_ significantly decreased (* *p* < 0.05 vs. control) the cell viability at all time points, and 24 h of treatment significantly (* *p* < 0.05 vs. control) reduced the cell viability at all tested concentrations (greyscale circles).

**Figure 2:**
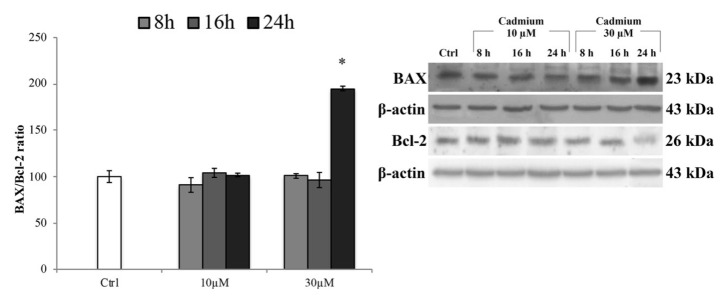
BAX and Bcl-2 protein expression levels. Representative western blot of the effects of CdCl_2_ (10 and 30 µM) on the protein levels of BAX and Bcl-2 after 8, 16, and 24 h of treatment. Bars represent the BAX/Bcl-2 ratio ± S.E.M., *n* = 9. Control condition was fixed at 100%; * *p* < 0.05 vs. control (untreated cells).

To ensure that this concentration did not induce death of endothelial cells triggering the apoptotic pathway (likely effect of acute exposure), we tested the expression levels of the pro-apoptotic protein BAX (Figure 2). The results showed that, at any time of exposure, a significant increase of BAX expression levels was not detectable, except for 30 µM at 24 h of treatment. These data were also corroborated by the analysis of cell morphology (Figure S1, supplementary materials). Therefore, also based on these results, we conducted the following experiments using 10 µM of Cd and exposure times of 8 and 16 h that failed to trigger cell death.

### 2.2. Cadmium-Dependent Alteration of BBB-Associated ZO-1 and Cytoskeletal Proteins

**Figure 3:**
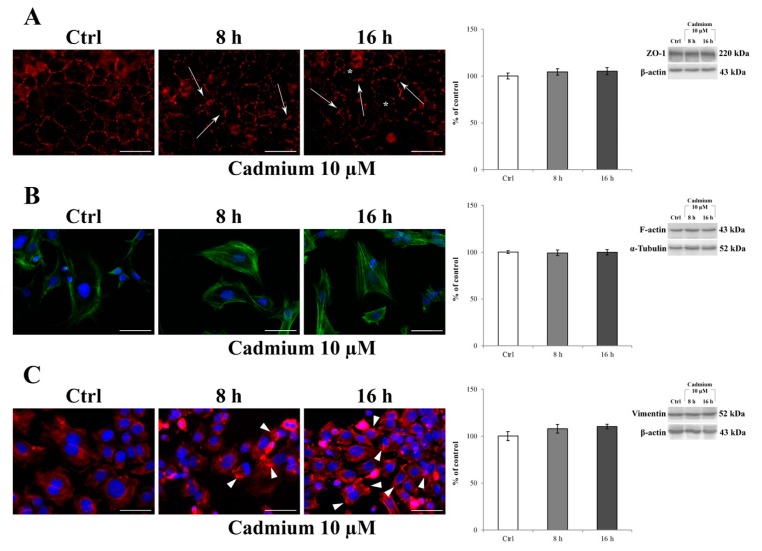
Effect of CdCl_2_ on ZO-1, F-actin, and vimentin localization in RBE4 cells. On the left: changes in the distribution of ZO-1 (red) (**A**), F-actin (green) (**B**), and vimentin (red) (**C**) in RBE4 cells treated with 10 μM CdCl_2_ for 8 and 16 h. Asterisks show holes formed between endothelial cells. In (**A**), arrows point to morphological alterations in intercellular junctions, indicative for loss of junctional function. In (**B**), note the Cd-dependent appearance of numerous stress fibers. In (**C**), Cd-dependent polarization of vimentin with the formation of clumps and aggregates (arrowheads) at the edges of the cell. Nuclei were stained with DAPI (blue). Total magnification 400×; *n* = 135; scale bar: 50 μm. On the right: representative western blot of the effects of CdCl_2_ on ZO-1, F-actin, and vimentin protein levels after 8 and 16 h of treatment. Bars represent the mean ± S.E.M., *n* = 4. Control condition was arbitrarily set at 100%.

Immunocytochemistry was used to assess the effect of 10 μM Cd treatment on the typical localization pattern of ZO-1, F-actin, and vimentin after 8 and 16 h of administration. Figure 3A shows that in control cells a ZO-1 marginal membrane localized to the cell–cell junctions, with a more prominent and clear immunostaining at the intercellular border (Figure 3A, control), which clearly suggests the presence of the physiological tightness of the barrier. Regarding the cytoskeletal proteins, F-actin exhibited its typical, marginal pattern of localization (Figure 3B, control), whereas vimentin appeared organized in thin fibers forming a network distributed throughout the cell cytoplasm and extending from the nucleus, where it formed a perinuclear ring (Figure 3C, control), to the periphery of the cell. The exposure of RBE4 cells to 10 μM Cd for 8 and 16 h caused time-dependent alterations in all the examined proteins; in particular, the following was evidenced: (1) a loss of ZO-1 staining, leading to a “zipper-like” staining pattern (Figure 3A, cadmium 10 µM, arrows) and holes that became visible between cells (Figure 3A, cadmium 10 µM, asterisks); (2) the formation of numerous F-actin stress fibers (Figure 3B, cadmium 10 µM), and (3) vimentin rupture, and stretching, as suggested by their straight alignment along the major cell axis (Figure 3C, cadmium 10 µM, arrowheads). These alterations were counteracted by the presence of 10 µM α-tocopheryl acetate in the medium (Figure S2), both at 8 and 16 h. Indeed, the ZO-1 alterations (Figure S2, panel A), F-actin stress fiber alterations (Figure S2, panel B), and vimentin aggregates as well (Figure S2, panel C) were superimposable with control, untreated cells. In addition, it is worth noticing that these morphological alterations were not attributable to a decrease in protein expression. Indeed, as reported by western blotting analysis (histograms on the right), ZO-1, F-actin, and vimentin expressions did not decrease after 10 µM Cd treatment both at 8 and 16 h.

### 2.3. Cadmium-Induced Overproduction of Intracellular ROS

**Figure 4:**
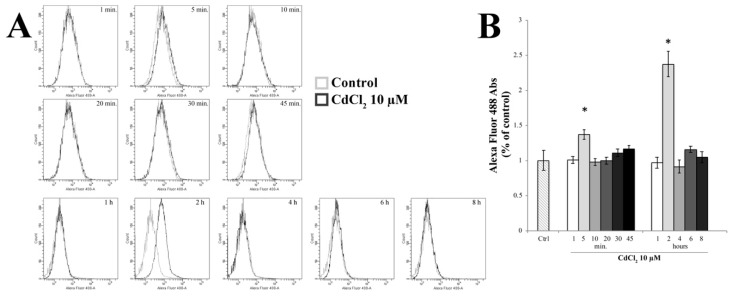
Evaluation of reactive oxygen species (ROS) generation. (**A**) Time-course (1–8 h) of ROS production by H_2_DCFDA fluorescence in control and in 10 µM Cd-treated RBE4 cells by fluorescence-activated cell sorting (FACS) analysis. (**B**) The reported values are expressed as mean ± S.E.M., *n* = 9. Control condition was arbitrarily set at 100%; * *p* < 0.05 vs. control (untreated cells).

Starting from previous data concerning the Cd-dependent oxidative effects [10], we wanted to evaluate the role of oxidative stress in TJ alterations by measuring intracellular ROS in RBE4 cells at different time points after 10 µM of Cd, using the redox-sensitive fluoroprobe dye CM-H_2_DCFDA by fluorescence-activated cell sorting (FACS) analysis, as previously reported [75]. As shown in Figure 4, an increase in ROS production was seen following incubation of cells with 10 µM Cd for 5 min, which decreased quickly to normalcy after 10 min of treatment (*p* < 0.05 vs. control), indicating that the increase of ROS generation was rapid and transient. However, the measurement of ROS after 2 h of treatment showed a further increase even more pronounced than that detected after 5 min of treatment.

### 2.4. Cadmium-Induced ER Stress in RBE4 Cells

**Figure 5:**
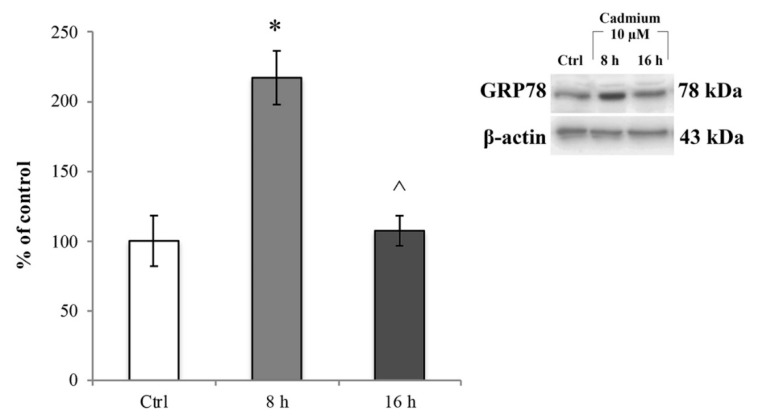
Cd-dependent endoplasmic reticulum (ER) stress. Immunoblot analysis and quantification of GRP78 expression at 8 and 16 h exposure to 10 μM Cd. Control condition was arbitrarily set as 100%, and results are expressed as mean ± S.E.M., *n* = 9. Significant at * *p* < 0.05 vs. control (untreated cells) and ^ *p* < 0.05 vs. 8 h treatment.

We then assessed whether the ROS increase induced ER stress. To this end, we investigated the changes in GRP78 protein expression levels, a chaperone well-known to be upregulated during ER stress [57]. RBE4 cells treated with 10 µM Cd for 8 and 16 h underwent immunoblot analysis on total cellular homogenates, and values were normalized to β-actin protein expression. As shown in Figure 5, 10 μM Cd significantly (* *p* < 0.05 vs. control) up-regulated GRP78 about three times compared to the control cells (Figure 5, light grey bar) at 8 h after administration. This effect was time-dependent because the protein expression decreased (Figure 5, dark grey bar) to values comparable to that of control cells at 16 h of Cd treatment. The effect of Cd on ER stress was fully counteracted by the presence of α-tocopheryl acetate in the medium at both time points (Figure S3, panels A and B). Taken together, these data indicated that after Cd treatment, RBE4 cells underwent ER stress following oxidative stress.

### 2.5. Cadmium Elicits Caspase-3 Activation

**Figure 6:**
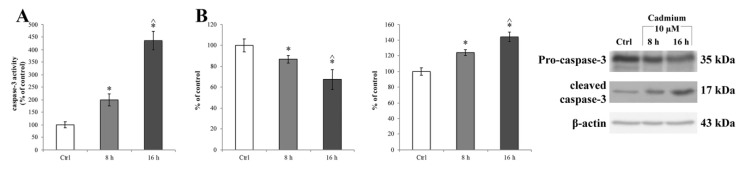
Caspase-3 activity in RBE4 cells after Cd treatment. (**A**) Caspase 3 activity, revealed by a fluorogenic peptide-based assay, and (**B**) upregulation of cleaved caspase-3 (17 kDa) and downregulation of pro-caspase-3 (35 kDa) revealed by western blot assay in RBE4 cells, post 10 μM Cd treatment for 8 and 16 h. β-actin was used as an internal control. The control condition was arbitrarily set as 100%, and results are expressed as mean ± S.E.M., *n* = 9. Significant at * *p* < 0.05 vs. control (untreated cells) and ^ *p* < 0.05 vs. 8 h treated cells.

Considering that ER stress can induce the activation of caspase-3 [76], we checked the activation of this downstream signal of ER stress and evaluated the caspase-3 enzymatic activity (Figure 6A) in the Cd-treated RBE4 cells using the EnzChek^®^ Caspase-3 Assay Kit (Molecular Probes, Milan, Italy). We found that Cd significantly (* *p* < 0.05 vs. control) activated caspase-3 at 8 h of treatment (Figure 6A, light grey bar). Also, Cd-dependent caspase-3 activation was confirmed by a western blot assay and showed an upregulation of the cleaved form of the protease complementary to a decrease of pro-caspase-3 (Figure 6B, light grey bar). Moreover, the time course of the protease activity did not parallel the decrease of GRP78 expression; indeed, at 16 h of treatment, caspase-3 activity was significantly elevated with respect to the control (* *p* < 0.05 vs. control) and of 8 h treated cells (^ *p* < 0.05 vs. 8 h treated cells). The effects of Cd on caspase-3 activation were counteracted by the presence of 10 µM α-tocopheryl acetate in the medium at both time points (8 and 16 h), as shown in Figure S4, panels A and B, respectively.

### 2.6. Cadmium-Dependent Extracellular ATP Release

The evidence of a Cd-dependent ATP release in renal cells [77] warranted us to characterize if RBE4 cells extruded ATP in response to Cd and the eventual the mechanisms through which it occurs.

**Figure 7:**
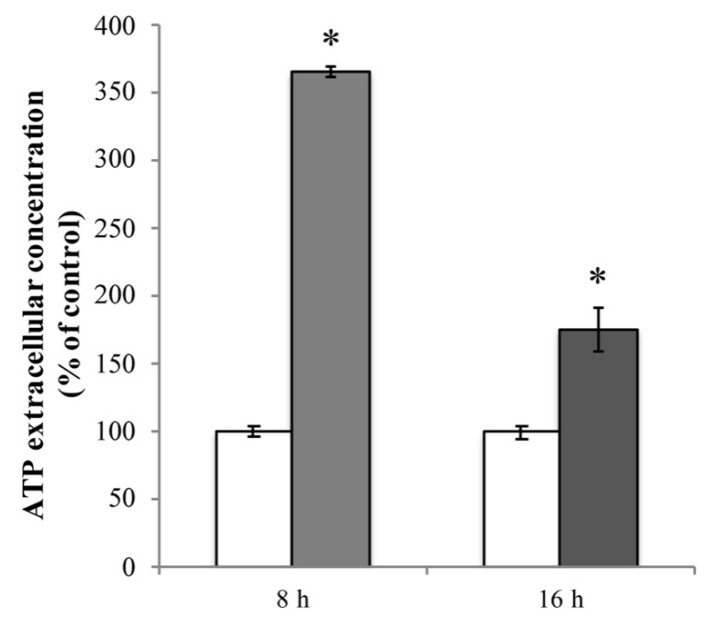
Cd-dependent release of ATP. Extracellular ATP release from RBE4 cells in the presence of 10 μM CdCl_2_ at 8 h (grey column) and 16 h (dark grey column). Control condition (white columns) was arbitrarily set as 100%, and results are expressed as mean ± S.E.M. *n* = 9, * *p* < 0.05 vs. control (untreated cells).

Firstly, we evaluated the extracellular ATP release in RBE4 cells that underwent 10 μM Cd treatment. In cultures exposed for 8 h to Cd, baseline ATP release was significantly elevated (* *p* < 0.05 vs. control) compared to control cells (Figure 7, light grey bar). This phenomenon was time-dependent since, after 16 h of treatment, the extracellular ATP levels, while remaining significantly higher with respect to untreated cells, exhibited a significant lowering tendency (^ *p* < 0.05 vs. 8 h treatment; Figure 7, dark grey bar).

### 2.7. Cadmium-Dependent ATP Release Is PANX1 Independent

**Figure 8:**
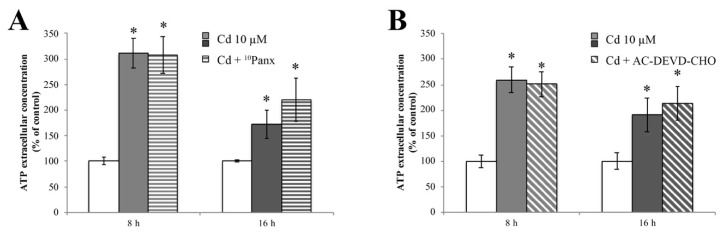
Release of ATP during Cd treatment is insensitive to ^10^Panx and AC-DEVD-CHO. ATP quantification assay performed in the presence of 100 μM of ^10^Panx (**A**), or 100 μM AC-DEVD-CHO (**B**), demonstrating that both PANX1 and caspase-3 inhibitors did not affect ATP release. Assays were performed in triplicate. Data are expressed as mean ± S.E.M., *n* = 10. The control condition (white columns) was arbitrarily set as 100%; * *p* < 0.05 vs. control (untreated cells).

Starting from the demonstration that RBE4 cells express PANX1 [75], and in order to verify if ATP release is dependent on its activation, we treated RBE4 cells with Cd for 8 and 16 h. ATP extrusion was measured in the presence and absence of the selective PANX1 inhibitor ^10^Panx. Results clearly showed that pretreatment with ^10^Panx did not result in significantly blunt Cd-dependent ATP release (Figure 8A).

Finally, we assessed whether caspase-3 induced PANX1 opening in RBE4 cells by evaluating ATP release after treatment with the AC-DEVD-CHO caspase-3 inhibitor. Results (Figure 8B) showed that the application of AC-DEVD-CHO did not attenuate ATP extrusion.

Since neither pharmacological inhibition of PANX1 nor caspase-3 inhibition prevented ATP spillage, we hypothesized that PANX1-mediated ATP release was not responsible for the ATP extrusion.

## 3. Discussion

Although studies in rodents have established Cd-dependent BBB dysfunction [30,32], how Cd may alter the cell–cell junctions in the endothelium remains elusive.

For this reason, we shed some light on the signaling pathway that underlies BBB endothelial cells alterations. The RBE4 cells preserve many features of the in vivo brain endothelium [74] and are widely accepted as an in vitro model for the study of the BBB [78]. In particular, these cells express the transporter for minerals, including the divalent metal transporter-1 (DMT-1) that nonselectively transports multiple divalent metals, including Cd, and mediates the cellular accumulation of the heavy metal [79]. By atomic absorption spectrophotometry, we confirmed that RBE4 cells accumulated Cd.

Moreover, accordingly to previously reported data in other cell lines [80,81,82,83], our results revealed that 10 μM CdCl_2_ induced an extensive alteration of the localization pattern of ZO-1 TJ protein as well as the cytoskeletal F-actin and vimentin proteins in RBE4 endothelial cells, indicating that TJs and associated proteins are primary targets of Cd. The choice to examine the Cd effects on ZO-1 depended on the fact that RBE4 cells are induced to synthesize and correctly localize occludin [84] and claudin-5 at the cell periphery and at the cell–cell junctions, only if endothelial cells are cocultured with neurons [79,85].

It has been reported that oxidative stress is a major component in the process of Cd-induced tissue injury [53,86]. Even if Cd does not directly generate free radicals, it has been demonstrated that it can indirectly increase intracellular accumulation of reactive oxygen species (ROS) and induce oxidative stress [87,88,89,90,91].

Thus, to delineate the underlying mechanism that triggers Cd-dependent TJ and cytoskeletal alterations, we examined ROS production. Our data showed that Cd induced a rapid ROS increase, reaching its peak at 5 min post-treatment with Cd, suggesting that the induction of an oxidative stress response is a very early event in the cellular reaction. The peak at 5 min was then rapidly followed by a decline of ROS levels that tended to disappear at 10 min after the challenge with Cd. Yet, we observed that ROS levels increased again after 2 h. The latter results are in agreement with previous work demonstrating that chronic exposure to Cd induced high levels of ROS and contributed to the acquisition of tolerance to the metal, as part of a defense mechanism to combat Cd-induced damages, including apoptosis [92,93,94,95]. Our observation of the lack of upregulation of the pro-apoptotic effector protein BAX further strengthened this result.

Given the notion that oxidative stress triggers ER stress [96], we assumed that ROS might be involved in Cd-induced ER stress; indeed, we observed that after 8 h of exposure to Cd, GRP78, a known unfolded protein response (UPR)-related chaperone [57,58,59,60,61], was highly upregulated. Thus, the GRP78-mediated ER stress response is involved in Cd-induced endothelial cells alterations. Also, the timing of these events strongly suggested that ROS could be the initiator of the ER stress. Furthermore, transient up-regulation of the GRP78 protein, here observed upon 16 h of Cd treatment, is in agreement with the cytoprotective role of this chaperone [97,98], supporting the role of ER stress in trying to maintain cell homeostasis [54,55,56].

On the other hand, it has been demonstrated that ER stress induces the activation of caspase-3 [99]. Although the production of caspase-3 is considered an apoptosis-specific event, evidence does exist that suggests a protective role also in the recognition and removal of overexpressed ROS [100,101,102,103]. In agreement with this view, we observed Cd-dependent activation of caspase-3 in RBE4 cells in the absence of a parallel activation of the apoptotic signaling cascade such as an increase in BAX protein expression levels. Moreover, the presence of 10 µM of α-tocopheryl acetate (a well-known antioxidant molecule [104]) prevented both the upregulation of GRP78 and caspase-3 cleavage during Cd treatment, confirming a prominent role of oxidative stress during the Cd-dependent signaling cascade.

Cd-dependent barrier dysfunctions are correlated with ATP depletion [62,63]. In vitro findings revealed that BBB damage is associated with an increase of extracellular ATP that, in turn, leads to alteration of tight junction components, such as ZO-1 [64]. Channels containing pannexin-1 (PANX1) are, among other routes, now widely accepted as putative mediators of ATP translocation to the extracellular milieu in non-excitable cells [105,106,107,108]. The recent demonstration that RBE4 cells expressed this channel [75] prompted us to investigate its role in ATP extrusion. The opening of PANX1 can occur via different mechanisms, one of which being the caspase-3-mediated cleavage of its autoinhibitory C-terminal domain [66]. Our data demonstrated that Cd induces an ATP release, and that this leakage is independent of the PANX1 opening and the proteolytic activity of caspase-3. This result is not entirely surprising since the caspase-3-mediated mechanism of PANX1 activation is closely related to the onset of the apoptotic pathway [109], a condition that did not occur in our experimental condition. It is then possible that the activation of caspase-3 in the early stage post Cd exposure might have a homeostatic role. The cellular mediator of ATP extrusion is currently under investigation.

In conclusion, even if the experiment in the presence of ROS inhibitors showed that all the events probably belong to the same signaling pathway, on the basis of our experiments, it is not possible to distinguish a cause–effect relationship. Further experiments are in progress to evaluate this point more exactly. Nevertheless, our data provide evidence that the interaction of Cd with the BBB vascular endothelium resulted in a dislocation of the TJ apparatus that, in turn, could result in secondary injuries to the CNS. Further investigation on the molecular mechanism underlying Cd-dependent BBB alterations could bring about new targets to develop specific therapies against Cd neurotoxicity.

## 4. Materials and Methods

### 4.1. Cell Culture and Treatments

The rat brain endothelial cell line RBE4 was kindly provided by Dr. Vincenzo Giuseppe Nicoletti (Department of Biomedical Sciences, University of Catania, Italy). As previously described [75], cells (up to passage 20) were grown and maintained in alpha-minimal essential medium (alpha-MEM)/Ham’s F10 (1:1) (GIBCO, Thermo Fisher Scientific, Milan, Italy) supplemented with 10% fetal bovine serum (FBS), 1% penicillin/streptomycin, 2 mM L-glutamine (EuroClone, Milan, Italy), and 1 ng/mL basic fibroblast growth factor (bFGF) (Thermo Fisher Scientific, Milan, Italy) at 37 °C and 5% CO_2_ in a humidified atmosphere. The growth medium was routinely changed 2–3 times per week. For each experimental setup, the cells were seeded on appropriate support for 24 h in complete growth medium until confluence, with this condition intended to mimic the intact BBB. Since Ca^2+^ and Cd^2+^ ions compete with each other for cell entrance [110], and the fixation of Cd^2+^ to serum proteins decreases the bioavailable ratio of the toxic cation [111], all treatments were performed in starvation medium. On the day of stimulation, the complete growth medium was replaced with starvation medium containing appropriate stimuli at different concentrations (CdCl_2_ and α-tocopheryl acetate).

^10^Panx (Proteogenix, Schiltigheim, France) was dissolved in 1% DMSO to obtain 1 mM solution and then diluted in physiological medium at a final concentration of 100 µM in the presence of Cd for 8 or 16 h. This concentration was chosen according to previously published data [112,113,114]. The caspase-3 inhibitor AC-DEVD-CHO (Calbiochem, Milan, Italy) was incubated for 1 h at a concentration of 100 µM before Cd treatment.

The rationale behind running experiments with α-tocopheryl acetate (a well-known antioxidant agent [115]) was to better elucidate the role of Cd-induced ROS in triggering the molecular pathway that leads to BBB alterations. On the other hand, ^10^Panx (the mimetic inhibitory peptide of PANX1 channel) and AC-DEVD-CHO (the caspase-3 inhibitor) were used to evaluate the role of ATP spillage in BBB alterations.

### 4.2. Analysis of Cellular Accumulation of Cd

Total Cd cellular accumulation was determined by atomic absorption spectrometry. RBE4 cells, at a density of 4 × 10^6^ cells, were plated in Petri dishes in complete growth medium. After 24 h, the cells were serum starved and stimulated with 10–30 µM CdCl_2_ for 24 h. After treatments, the medium was removed, and cells were washed and then scraped in cold phosphate-buffered saline (PBS). The cell suspensions were centrifuged at room temperature (RT) for 10 min at 1000 rpm. The pellets obtained were washed again in PBS and centrifuged at RT for 10 min at 1000 rpm. The pellets obtained (about 300 mg for each experimental point) were immediately stored at −80 °C and subsequently lyophilized. Freeze-dried material (about 30 mg) was mineralized with 10 mL of 65% HNO_3_ (Panreac Applichem, ITW Reagents) at 200 °C for 20 min using a microwave digestion system (Mars 6, CEM). After digestion, the volume was adjusted to 25 mL with milliQ water, and Cd concentrations in the solutions were determined by an atomic absorption spectrophotometer (AAnalyst 200, Perkin Elmer, Waltham, MA, USA). Certified reference material (grade BCR, Fluka Analytical, Sigma–Aldrich, Milan, Italy) was used to verify the accuracy and the precision of the method. Total cellular accumulation of Cd was expressed as micrograms per gram of cellular pellet dry weight. Cadmium accumulation in control and Cd-exposed cells was analyzed in three separate culture preparations (*n* = 3).

### 4.3. MTT Assay

Cell viability was evaluated by the reduction of 3-(4,5-dimethylthiozol-2-yl)-2,5-diphenyltetrazolium bromide (MTT) by mitochondrial dehydrogenase, which directly reflects the activity of mitochondria, and can be considered an indirect measurement of cell viability. RBE4 cells were plated into 96 multiwell plates, at a density of 2.5 × 10^4^ cells/well, in complete growth medium. The following day, the cells were starved as described above and treated with different concentrations of Cd (1, 3, 10, 30, and 100 µM) for 8, 16, and 24 h. After treatment, the starvation medium containing stimuli was removed and substituted with 1 mg/mL MTT (in starvation medium without phenol red). The chromogenic solution was incubated for at least 20 min at 37 °C. The formazan crystals that formed were dissolved by adding 100 μL of DMSO in each well, and the absorption at 570 nm was read using a microplate reader (MultiskanFC™ microplate photometer, ThermoFisher Scientific, Milan, Italy). Each experiment was performed five times, in quintuplicate.

### 4.4. Observation of Cell Morphology

RBE4 cells were plated at a density of 10^6^ cells in 60 mm Petri dishes. At 90% confluence, cells were treated with 10 or 30 μM CdCl_2_ for 8 and 24 h. Cell morphology was observed with an inverted light microscope (XDS-2, Optika, Bergamo, Italy), and a representative picture was captured with ThrueChrome HD II (TiEsseLab S.r.l., Milan, Italy) at a total magnification of 100×.

### 4.5. Western Blotting

RBE4 cells, at a density of 3.5 × 10^6^ cells, were plated in Petri dishes in complete growth medium. After 24 h, the cells were serum starved and stimulated with 10–30 µM Cd for 8, 16, and 24 h with or without α-tocopheryl acetate 10 µM (at 8 and 16 h). After treatments, the medium was removed, and cells were scraped in cold PBS. The cell suspensions were centrifuged at RT for 10 min at 1000 rpm. The pellets obtained were treated with a Mem-PER™ Plus Membrane Protein Extraction Kit (Thermo Fisher Scientific, Milan, Italy), following the manufacturer’s instructions, to isolate the integral and attached membrane proteins isolated from the cytosolic proteins. The protein concentration was evaluated by the Bradford method, and equal amounts of protein (15 µg) were separated on a 12% polyacrylamide gel by electrophoresis and transferred onto a nitrocellulose membrane (Porablot NPC, MACHEREY-NAGEL, Milan, Italy). After 1 h blocking with 3% bovine serum albumin (BSA) in Tris-buffered saline containing 0.1% Tween 20 (T-TBS) at RT, the blots were incubated in T-TBS/3% BSA overnight at 4 °C with the following primary antibodies: rabbit primary antibody for GRP78 (ThermoFisher Scientific, Milan, Italy) at 1:500 dilution, for cleaved caspase-3 (Cell Signalling, Euroclone, Milan, Italy) at 1:1000 dilution, and for BAX (Santa Cruz Biotechnology, Milan, Italy) at 1:300 dilution, mouse primary antibody for pro-caspase-3 (Santa Cruz Biotechnology, Milan, Italy) at 1:500 dilution, and for Bcl-2 (EMD Millipore, Milan, Italy) at 1:1000 dilution. After washing with T-TBS, the goat anti-rabbit/mouse HRP secondary antibodies (Santa Cruz Biotechnology) were added at 1:5000 dilution in T-TBS for 1 h at RT and then washed again. Proteins were detected with the Amersham ECL Plus Western Blotting Detection Reagent (GE Healthcare, Milan, Italy). Protein expression levels were then quantified by ImageJ analysis software (ImageJ, National Institute of Health, USA, http://imagej.nih.gov/ij, 1.47t). β-actin (1:10,000 dilution) (Santa Cruz Biotechnology) normalization was performed for each sample. Each experiment was performed three times, in triplicate.

### 4.6. ZO-1 Tight Junction, F-Actin, and Vimentin Immunofluorescent Labeling

In order to investigate Cd-induced BBB morphological alterations, we investigated the effects of Cd on the ZO-1 complex, which plays a critical role in anchoring the TJ proteins to the cytoskeletal apparatus [42]. Furthermore, since ZO-1 is directly linked to the actin cytoskeleton [45,46,47], filamentous actin microfilament (F-actin) and the intermediate filament vimentin, which are the major constituents of the cytoskeleton that establishes inter-endothelial junctional integrity, defining the peripheral morphological belt of endothelial cells [48,49], and connecting the nuclear periphery to the inner surface of the plasma membrane [50], we used these proteins as markers of tight junction formation in the monolayers of brain microvascular endothelial cells as previously reported [43].

Briefly, to visualize the distributions of ZO-1, F-actin, and vimentin, 1.5 × 10^5^ RBE4 cells were seeded on sterilized coverslips (lodged in a 6 multiwell plate) in complete growth medium. After 24 h, cells were serum starved and stimulated with 10 µM Cd for 8 and 16 h with or without 10 µM α-tocopheryl acetate. Cells were fixed with 4% formaldehyde dissolved in PBS for 10 min at RT for F-actin and vimentin, and with cold methanol for 20 min for ZO-1. After permeabilization for 10 min with 0.1% TritonX-100, cells were incubated in 1% BSA in PBS for 45 min to block nonspecific antibody binding, and then they were incubated with Alexa-488 conjugated phalloidin (1:200; ThermoFisher Scientific, Milan, Italy) to stain actin or with mouse anti-vimentin antibody (1:100) and with rabbit anti-ZO-1 antibody (1:50) overnight at 4 °C. Vimentin and ZO-1 primary antibody were visualized by incubation with an Alexa Fluor 568 goat anti-mouse or anti-rabbit IgG secondary antibody respectively (1:200; Invitrogen, Milan, Italy) conjugated with Alexa Fluor 568 for 1 h at RT. After counterstaining with DAPI (4′,6-diamidin-2-fenilindolo; 1:2000 dilution; Invitrogen, Milan, Italy) for 5 min at RT, coverslip glasses were mounted using Fluoromount antifade solution (ThermoFisher Scientific, Milan, Italy) on cover slides. Fluorescent signals were detected at 400× total magnification (five microscopic fields for each experimental point) by a motorized Leica DM6000B microscope equipped with a DFC350FX camera. Negative controls were performed by omitting the primary antibody to confirm the specificity of primary antibodies and by omitting the secondary antibody to reveal autofluorescent labeling (data not shown).

### 4.7. Quantification of Intracellular ROS by Flow Cytometry

RBE4 cells were plated in Petri dishes using complete growth medium, reaching about 80% confluence. After that, the cells were starved and stimulated with 10 µM of Cd from 1 min to 8 h. After each treatment, cells were washed twice with DMEM without phenol red and detached from Petri dishes by trypsin/EDTA and centrifuged at 1000 rpm for 5 min at RT. The pellets were gently resuspended in DMEM without phenol red and labeled with 1 µM CM-H_2_DCFDA (Life Technologies, ThermoFisher Scientific, Milan, Italy). The tubes were gently mixed and incubated in the dark at 37 °C for 20 min. After labeling, cells were centrifuged again, the supernatant was discarded, and the pellets obtained were gently resuspended in DMEM without phenol red and immediately analyzed using a FACS flow cytometer (Becton–Dickinson, San Jose, CA, USA).

The sample flow rate was adjusted to about 103 cells/s. For a single analysis, the fluorescence properties of about 2.5 × 10^4^ RBE4 cells were collected. Each experiment was performed three times, in triplicate.

### 4.8. Caspase-3 Enzymatic Activity

RBE4 cells were plated in 6-well plates (5 × 10^5^/well) using complete growth medium. The following day, cells were starved and stimulated with 10–30 µM Cd for 8 and 16 h. After treatment, cells were scraped in 100 mM lysis buffer (200 mM Tris-HCl buffer, pH 7.5, containing 2 M NaCl, 20 mM EDTA, and 0.2% TritonX-100). Fifty microliters of the supernatant was incubated with 25 µM fluorogenic peptide caspase-3 substrate rhodamine 110 bis-(*N*-CBZ-l-aspartyl-l-glutamyl-l-va- lyl-l-aspartic acid amide) (AC-DEVD-CHO; Molecular Probes) at 25 °C for 30 min. The amount of cleaved substrate in each sample was measured in a 96-well plate fluorescent spectrometer (Perkin-Elmer; excitation at 496 nm and emission at 520 nm). Each experiment was performed three times, in triplicate.

### 4.9. Extracellular ATP Quantification

RBE4 cells were plated in 6-well plates (5 × 10^5^/well) using complete growth medium. The following day, cells were starved and stimulated with 10–30 µM Cd for 8 and 16 h. After treatment, the medium was harvested, and 50 µL was processed following the manufacturer’s procedure (ATPlite Luminescence ATP Detection Assay System, PerkinElmer Italia, Milan, Italy). The extracellular ATP was quantified by luminescence using a VICTOR microplate reader (PerkinElmer, Milan, Italy). Each experiment was performed three times, in triplicate.

### 4.10. Statistical Analysis

Each experiment was performed at least three times, and data were expressed as mean ± SEM. Multiple-sample comparisons were carried out on the raw data by one-way ANOVA followed by a Student’s *t*-test. In all comparisons, differences were considered significant when the *p* value was less than 0.05 (*p* < 0.05) and 0.01 (*p* < 0.01). All statistical analyses were performed using Origin 9 statistical analysis software (OriginLab, Northampton, USA).

## Supplementary Materials

Supplementary materials can be found at https://www.mdpi.com/1422-0067/20/23/6010/s1.
