# Supplementary material for: Effects of Cadmium on ZO-1 Tight Junction Integrity of the Blood Brain Barrier
Source: Int J Mol Sci. 2019 Nov 29;20(23):6010. doi: 10.3390/ijms20236010 (PMC6928912; doi:10.3390/ijms20236010)
Supplement: Supplementary file 1 [file ijms-20-06010-s001.pdf]

Supplemental figures:

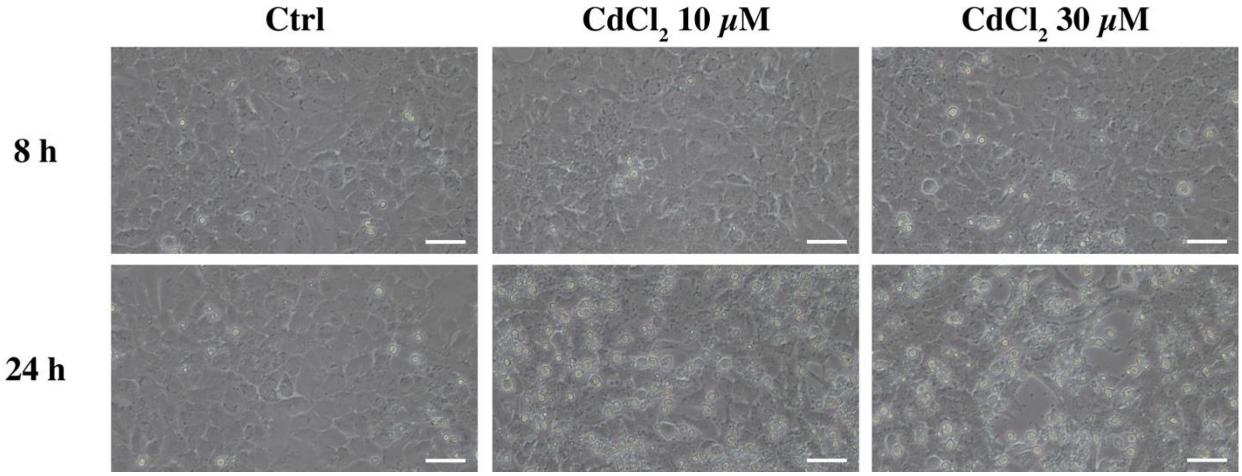

Figure S1. Cd-induced cytotoxicity in RBE4 cells. Morphologic alterations in RBE4 cells treated with $CdCl_2$ 10 μM and 30 μM for 8 and 24 hours. Images were taken with an Optika XDS-2 inverted phase contrast microscope equipped with a TrueChrome HDII camera. Total magnification 100X. Scale bar: 50 μm.

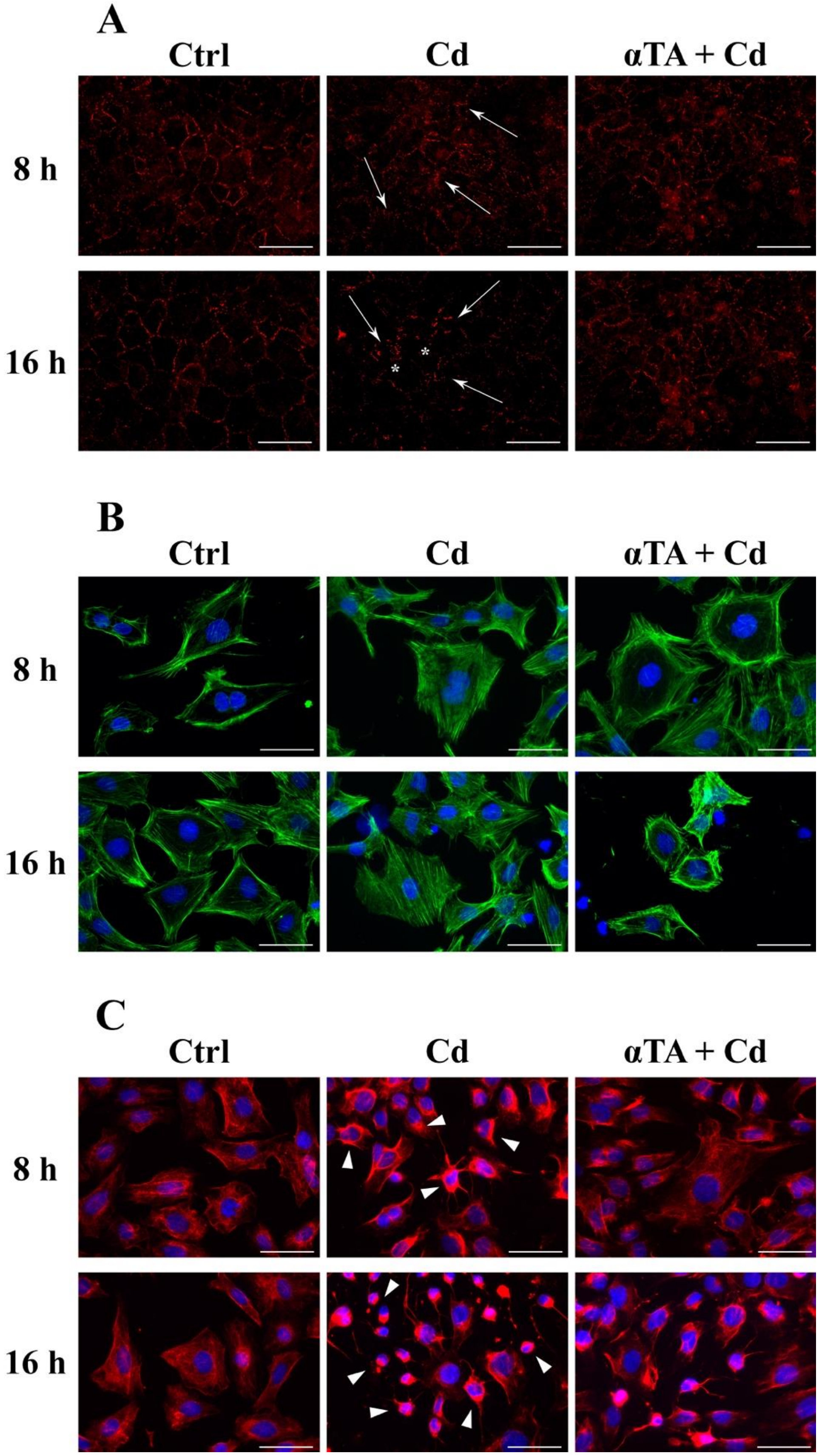
A
Ctrl
Cd
αTA + Cd
8 h
16 h
B
Ctrl
Cd
αTA + Cd
8 h
16 h
C
Ctrl
Cd
αTA + Cd
8 h
16 h

Figure S2. αTocopheryl acetate counteracts $CdCl_2$ effects on ZO-1, F-actin and vimentin localization. The changes induced by $CdCl_2$ 10 μM, both at 8 and 16 h, in the distribution of ZO-1 (**A**), F-actin (**B**), and vimentin (**C**) in RBE4 cells, was clearly counteracted by the presence of αTA 10 μM. In panel **A** it is important to note the holes (asterisks) formed between endothelial cells, and the morphological alterations in intercellular junctions (arrows), indicating the loss of junctional function. These alterations are counteracted by the presence of αTA during $CdCl_2$ treatment. Panel **B** show the absence of stress fibers during the αTA treatment in presence of $CdCl_2$. In panel **C**, αTA presence, both at 8 and 16 h, clearly show a decrease in vimentin aggregates and clumps (arrowheads). Total magnification 400X, n=135; bar: 50 μm.

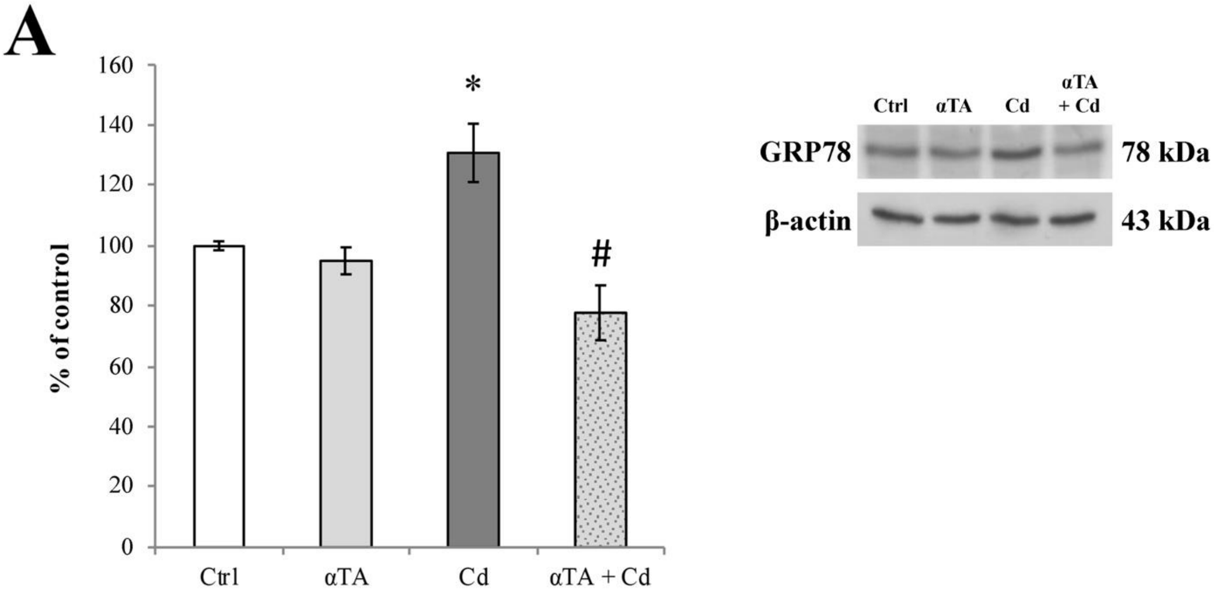

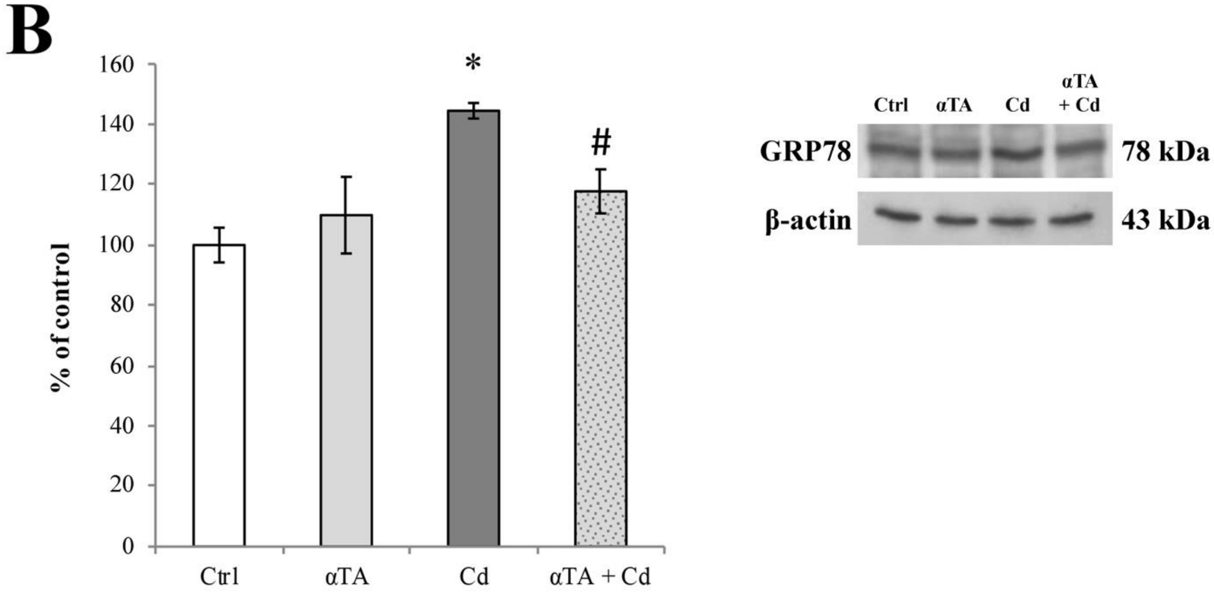

Figure S3. αTocopheryl acetate counteracts the Cd-induced ER stress. Western blotting analysis reveal that αTA 10 μM counteract the Cd-induced upregulation of GRP78 expression both at 8 (panel A) and 16 h (panel B). Control condition was arbitrarily set as 100 % and results are expressed as mean ± S.E.M., n=9; *p< 0.05 *vs* control (untreated cells); #p< 0.05 *vs* Cd.

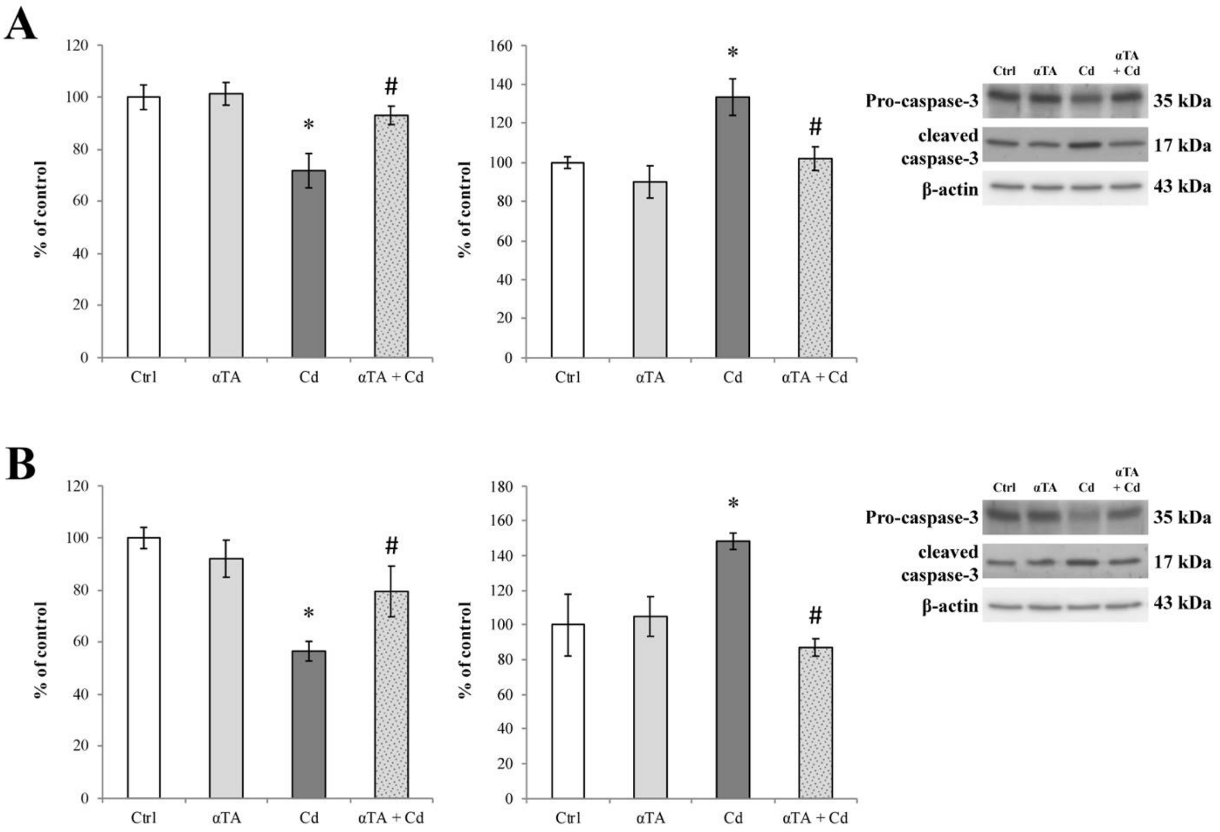

Figure S4. αTocopheryl acetate counteracts the Cd-induced caspase-3 activation. Western blotting analysis show that αTA 10 μM counteract the Cd-induced caspase-3 activation, both at 8 (panel A) and 16 h (panel B). Control condition was arbitrarily set as 100 % and results are expressed as mean ± S.E.M., n=9; *p< 0.05 *vs* control (untreated cells); #p< 0.05 *vs* Cd.
